# The deadliest snake according to ethnobiological perception of the population of the Alto Juruá region, western Brazilian Amazonia

**DOI:** 10.1590/0037-8682-0305-2019

**Published:** 2019-12-20

**Authors:** Jessyca Lima da Silva, Ageane Mota da Siva, Gardênia Lima Gurgel do Amaral, Givanildo Pereira Ortega, Wuelton Marcelo Monteiro, Paulo Sérgio Bernarde

**Affiliations:** 1 Universidade Federal do Acre, Campus Floresta, Centro Multidisciplinar, Laboratório de Herpetologia, Cruzeiro do Sul, AC, Brasil.; 2 Universidade Federal do Acre, Programa de Pós-Graduação Stricto Sensu em Ciências da Saúde na Amazônia Ocidental, Rio Branco, AC, Brasil.; 3 Instituto Federal do Acre, Campus de Cruzeiro do Sul, Cruzeiro do Sul, AC, Brasil.; 4 Universidade Federal do Acre, Programa de Pós-Graduação Bionorte, Rio Branco, AC, Brasil.; 5 Universidade do Estado do Amazonas, Manaus, AM, Brasil.; 6 Fundação de Medicina Tropical Dr. Heitor Vieira Dourado, Manaus, AM, Brasil.

**Keywords:** State of Acre, Snakes, Snakebites, Ophidism, Envenomation

## Abstract

**INTRODUCTION:**

We examined the ethnobiological perception of the population of the Alto Juruá region about different snake species, in terms of their dangerousness and manifestations of envenomation.

**METHODS:**

We interviewed 100 villagers who were active in the forests.

**RESULTS:**

*Lachesis muta* was considered the most venomous snake, and *Bothrops atrox* appeared to be the most feared snake species.

**CONCLUSIONS:**

The high incidence, severity, and mortality of *B. atrox* bites and the severity and mortality of *L. muta* bites were the factors that contributed to these species being perceived as the most feared and venomous snakes.

Snakebites do not have epidemic potential like infectious and vector-borne parasitic diseases, however, the annual global mortality due to snake envenomation is by far higher than that attributed to several currently neglected tropical diseases including dengue hemorrhagic fever, cholera, leishmaniasis, schistosomiasis, Japanese encephalitis, and Chagas disease^1^. Snakebites are thus considered an important issue regarding incidence and severity, and the clinical manifestations of snakebites encouraged the World Health Organization to recognize and include snakebites in the category of Neglected Tropical Diseases in 2017^2^. It is estimated that globally up to 5,500,000 snakebites occur per year, which result in 1,841,000 cases of envenomation and 94,000 deaths^3^. The most vulnerable victims are typically members of the poorest communities living in rural areas of various countries in Africa, Asia, and Latin America^1^ ^,^ ^2^. 

In Brazil, snakebites are mainly associated with activities in agriculture, and in the Amazon region, beside extractive activities, there are also people living in forests (extractivist, riverine, indigenous)^4^ ^,^ ^5^ ^,^ ^6^ ^,^ ^7^. In order to assess which snake is considered the most dangerous species, the number of deaths caused by each species and the severity of bites must be taken into account^8^. There are four groups of venomous snakes in Brazil, of which the genus *Crotalus* is considered the most dangerous with 0.96% lethality, followed by the genus *Lachesis* (0.61% lethality), the genus *Bothrops* (0.37%), and the genus *Micrurus* (0.27%)^9^. Bothropic and lachetic envenomations are the main causes of morbidity and mortality associated with snakebites in the Brazilian Amazon region: 67% of reported snakebites and 65.8% of snakebite-related deaths are attributed to the genus *Bothrops*, and the genus *Lachesis* is reported to be responsible for 21.8% of snakebites and for 29.5% of snakebite-related deaths^6^ ^,^ ^10^. Therefore, although snakes of the genus *Bothrops* appear to bite more frequently, the species *L. muta* is associated with the highest lethality. 

In Alto Juruá, a region in the western Brazilian Amazonia, snakebites are considered an important issue of which predominantly communities in rural areas and forests are affected^4^ ^,^ ^7^ ^,^ ^11^. We describe here the ethnobiological perception of dangerousness of different snake species by the population of the rural areas of Alto Juruá regarding. Moreover, we summarize clinical manifestations of bites by the mentioned snake species.

We conducted a transversal study with consecutive data collection from March to April 2019. We interviewed people living near the forest close to the lower Moa River and who pursued activities such as extractivism, fishing, and hunting in forests in the region located in the municipality of Cruzeiro do Sul, Alto Juruá, in the west of the state of Acre. The predominant activities of the local population are fishing, fish farming, manioc production, extractivism, and crop farming^7^.

Interviews were conducted individually and anonymously using a semi-structured script based on several previously chosen topics, which facilitated collecting large amounts of information with minimum bias^12^. Members of the community were interviewed if they pursued any activity in the forests on a regular basis. After each interview, the interviewee was asked to indicate other people who also frequented the forests. The following three questions were asked: 1) do you know what happens to a person when they are bitten by one of these snakes: jararaca, surucucu, papagaia, pico de jaca, and coral, 2) which of these is the most venomous, and 3) which of these are you most afraid of? The regionally used common names correspond to species involved in snakebites in the region^7^ ^,^ ^10^ ^,^ ^11^: jararaca - juvenile *B. atrox*, surucucu - adult *B. atrox*, papagaia - *B. bilineatus smaragdinus*, pico-de-jaca - *L. muta*, and coral - *Micrurus* spp. The consequences of snakebite were recorded exactly as reported, according to the respective dialect, and were re-phrased in a more technical manner here. Re-phrasing included terms such as "rotting" (necrosis), "swelling" (edema), "crippling" (amputation), "blood coming out through the nose" (epistaxis), "spitting blood" (gingival bleeding), and “coughing up blood" (hemoptysis).

This study is part of the project "The Ethnoherpetology Study in Alto Juruá - Acre", approved by the Ethics Review Board for Research with Human Subjects at the União Educacional do Norte Ltda - UNINORTE, Rio Branco (approval number: 2,092,523).

One-hundred people aged 13-91 years (mean age 43 years) were interviewed (79 men and 21 women; Table 1). *L. muta* snakes were considered most venomous (by 61%), and *B. atrox* was the most feared snake (by 47%; Table 1). Regarding consequences of snakebites, people knew most about juvenile and adult *B. atrox* (85% and 84% answered, respectively), followed by *L. muta* (62%), *B. bilineatus smaragdinus* (46%), and *Micrurus* spp. (24%; Table 2; Figure 1). The respective predominant symptoms or consequences were reported as follows - juvenile *B. atrox*: bleeding, pain, and edema; adult *B. atrox*: pain, edema, and dizziness; *B. b. smaragdinus*: pain, edema, and thirst; *L. muta*: amputation, death, and pain; *Micrurus* spp.: pain, edema, headache, and thirst (Table 2). Regarding hemorrhages caused by juvenile *B. atrox*, interviewees described different forms of bleeding: through the pores of the skin (24%), at the location of the bite (19%), gingival bleeding (15%), through hair roots (15%), from all orifices (2%), through the nose (1%), hematemesis (1%), otorrhagia (1%), hematuria (1%), hemoptysis (1%), and from the fingernail beds (1%).

**TABLE 1:** Perception of the peoples of the lower Moa River region (Cruzeiro do Sul - AC) on the dangerousness of venomous snakes.

Perception regarding snakes	Frequency of answers
MOST VENOMOUS SNAKE	
Pico de jaca (*Lachesis muta*)	61%
Surucucu (adult *Bothrops atrox*)	31%
Jararaca (juvenile *Bothrops atrox*)	13%
Papagaia (*Bothrops bilineatus smaragdinus*)	2%
Coral (*Micrurus* spp.)	2%
MOST FEARED SNAKE	
Surucucu (adult *Bothrops atrox*)	47%
Pico de jaca (*Lachesis muta*)	40%
Jararaca (juvenile *Bothrops atrox*)	16%
Papagaia (*Bothrops bilineatus smaragdinus*)	5%
Coral (*Micrurus* spp.)	2%
All types	4%
None	4%

**TABLE 2:** Consequences of snakebites according to the peoples of the lower Moa River region (Cruzeiro do Sul - AC).

	**juvenile *B. atrox***	**adult *B. atrox***	*B. bilineatus smaragdinus*	*L. muta*	***Micrurus* spp.**
Regional common name	jararaca	surucucu	papagaia	pico de jaca	coral
Percentage of people who responded	85%	84%	43%	62%	24%
POTENTIAL CONSEQUENCES					
Pain	29%	35%	15%	21%	9%
Edema	23%	31%	12%	13%	8%
Hemorrhage	64%	15%	7%	4%	4%
Necrosis	2%	14%	0%	8%	0%
Headache	23%	16%	8%	5%	5%
Dizziness	17%	21%	6%	9%	4%
Blurred vision	17%	18%	5%	14%	3%
Thirst	19%	19%	8%	11%	5%
Vomiting	7%	13%	5%	3%	1%
Paresthesia	4%	2%	3%	3%	0%
Death	0%	4%	6%	24%	3%
Amputation	0%	8%	0%	25%	2%
Muscular atrophy	2%	5%	3%	4%	0%
Sweating	3%	1%	1%	0%	1%
Fever	3%	4%	0%	2%	2%
Bruising	2%	2%	0%	0%	0%
Change in heart rate	2%	1%	0%	0%	2%
Nausea	2%	3%	2%	1%	1%
Fainting	3%	11%	2%	6%	1%
Loss of speech	3%	5%	1%	0%	0%
Burning	1%	2%	0%	1%	0%
Erythema	1%	0%	0%	0%	0%
Blisters	0%	1%	0%	0%	0%
Scarring	0%	4%	0%	0%	0%
Mental confusion	1%	3%	2%	2%	0%
Weakness	1%	1%	2%	1%	0%
Fear	1%	1%	0%	0%	0%
Distress	1%	0%	0%	0%	0%
Infection	0%	1%	0%	0%	0%
Venom rising through the body	0%	1%	0%	0%	0%
Hypersalivation	0%	0%	0%	1%	0%
Hoarseness	0%	0%	0%	1%	0%
Breathing difficulties	0%	0%	0%	1%	0%

**FIGURE 1:**
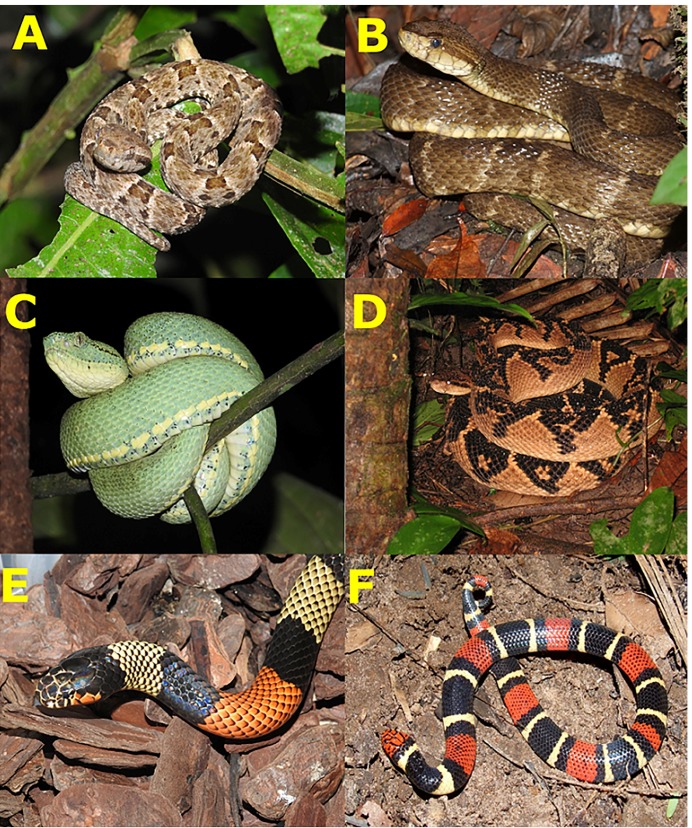
A) Jararaca (juvenile *Bothrops atrox*); B) Surucucu (adult *B. atrox*); C) Papagaia (*B. bilineatus smaragdinus*); D) Pico de jaca (*Lachesis muta*); E) Coral (*Micrurus spixii*); F) Coral (*M. surinamensis*). Photos: Paulo Bernarde.

*L. muta*, which causes the highest lethality, was considered the most venomous species due to the severity of its bites with 25-24% of amputations and deaths, respectively. Bites by the genus *Lachesis* occur infrequently, and *L. muta* snakes inhabit regions of low population density^13^, however, this species is commonly known to be dangerous^5^, probably due to the severity of its bite and because of its large size which can exceed three meters. 

Despite not being considered the most venomous snake, *B. atrox* was regarded as the most feared snake in this study, particularly adult individuals, regionally termed surucucu^7^. This may be because this species is responsible for the most snakebites with the highest morbidity and mortality^10^. *B. atrox* was most frequently reported to cause pain (35% of the interviewees), edema (31%), and necrosis (14%), all of which is typically associated with its bites^10^ ^,^ ^13^ ^,^ ^14^. In a study conducted at the Baixo Purus river in the Amazon region, *B. atrox* and *L. muta* snakes were also the species that were most feared by the riverine population^5^. At the Baixo Purus river, *L. muta* was considered less aggressive than *B. atrox* ^5^, which is less aggressive may therefore have been considered the second most-feared species by the subjects in the present study.

Most reported consequences of snakebites (pain, edema, hemorrhage, necrosis, headache, dizziness, blurred vision, thirst, vomiting, paresthesia, death, amputation, muscular atrophy, sweating, fever, bruising, changes in heart rate, nausea, fainting, burning, erythema, blisters, scarring, weakness, infection, hypersalivation and shortness of breath) may indeed occur^4^ ^,^ ^6^ ^,^ ^10^ ^,^ ^13^ ^,^ ^14^, however, the consequence was not always attributed to the correct species by the interviewees. Several reported consequences seemed not to be physiologically related to envenomation, but rather to psychological reactions (mental confusion, fear, and distress) or originate from misinformation (loss of speech and the assumption that "venom rises through the body"). Symptoms caused by *B. atrox* were reported more accurately than those cause by other species, particularly regarding those caused by *L. muta* and *Micrurus* spp., probably because *B. atrox* bites occur considerably more frequently in this region than bites by other species^4^ ^,^ ^7^ ^,^ ^11^. Juvenile *B. atrox* feed predominantly on amphibians and lizards, whereas adults preferably prey on rodents, and this dietary change may be associated with differences in venom composition^15^. Juvenile *B. atrox* inject a smaller amount of venom per bite, however, their bites may cause prominent vasculotoxic effects such as hemorrhage and edema^15^. Juvenile and adult specimens of *B. atrox* are perceived as belonging to different species^7^, and interviewees differentiated between the effects of bites by juveniles (more hemorrhagic) and adults (increased probability of necrosis) and detailed potential forms of bleeding (e.g., local bleeding, nose bleeds, hematemesis, otorrhagia, hematuria, and hemoptysis^10^ ^,^ ^13^ ^,^ ^14^).

All species of venomous snakes that occur in the Alto Juruá region can cause death^10^, however, several factors need to be considered regarding the outcome of a snakebite (e.g., elapsed time between envenomation and serum therapy, age and bodyweight of the victim, species and size of the snake, amount of venom, anatomical region of the bite, quality of health care, co-morbidity^6^ ^,^ ^8^ ^,^ ^9^ ^,^ ^10^). Several snakes which produce comparably stronger venom may cause fewer accidents when population density and their propensity to bite are taken into account and may thus be responsible for fewer deaths in a given region than other snakes^8^. *L. muta* is responsible for comparably few snakebites, and it is a rare species in forest environments, however, the severity of its bites^10^seems to have given rise to its reputation as most venomous species in this region. In contrast, *B. atrox* is the most abundant species of venomous snakes in various environments such as forests, crop fields, and pastures, and it is responsible for a larger proportion of snakebite-related morbidity and mortality in the Amazon region^4^ ^,^ ^7^ ^,^ ^10^ ^,^ ^11^ ^,^ ^14^, which is why this species was the most feared snake in the Alto Juruá region and was considered the most dangerous one.

Local people were more aware of the effects of *B. atrox* bites, including differences between bites by juvenile and adult specimens, probably because it is the most abundant venomous snake species in the Alto Juruá region. The lower frequency of bites by other species probably explains the lack of knowledge on the respective effects. Regarding morbidity, mortality, and severity, *B. atrox* is the most important venomous snake in the Brazilian Amazon^4^ ^,^ ^6^ ^,^ ^7^ ^,^ ^10^ ^,^ ^13^ ^,^ ^14^ and, in this study, this species was the most feared snake by the residents of Alto Juruá. In comparison, *L. muta* was considered the most venomous snake in this region, probably due to the severity and lethality of its bites.
